# Eosinophils as drivers of bacterial immunomodulation and persistence

**DOI:** 10.1128/iai.00175-24

**Published:** 2024-07-15

**Authors:** Katelyn M. Parrish, Monica C. Gestal

**Affiliations:** 1 Department of Microbiology and Immunology, Louisiana State University Health Sciences Center at Shreveport, Shreveport, Louisiana, USA; Department of Microbiology and Environmental Toxicology, University of California at Santa Cruz, Santa Cruz, California, USA

**Keywords:** whooping cough, pertussis, *Bordetella *spp., BtrS, eosinophils, mucosal immunity, immune homeostasis, Th17 microenvironment, long-term protection, persistence, mucosal infections

## Abstract

Traditionally, eosinophils have been linked to parasitic infections and pathological disease states. However, emerging literature has unveiled a more nuanced and intricate role for these cells, demonstrating their key functions in maintaining mucosal homeostasis. Eosinophils exhibit diverse phenotypes and exert multifaceted effects during infections, ranging from promoting pathogen persistence to triggering allergic reactions. Our investigations primarily focus on *Bordetella* spp., with particular emphasis on *Bordetella bronchiseptica*, a natural murine pathogen that induces diseases in mice akin to pertussis in humans. Recent findings from our published work have unveiled a striking interaction between *B. bronchiseptica* and eosinophils, facilitated by the *btrS*-mediated mechanism. This interaction serves to enhance pathogen persistence while concurrently delaying adaptive immune responses. Notably, this role of eosinophils is only noted in the absence of a functional *btrS* signaling pathway, indicating that wild-type *B. bronchiseptica*, and possibly other *Bordetella* spp., possess such adeptness in manipulating eosinophils that the true function of these cells remains obscured during infection. In this review, we present the mounting evidence pointing toward eosinophils as targets of bacterial exploitation, facilitating pathogen persistence and fostering chronic infections in diverse mucosal sites, including the lungs, gut, and skin. We underscore the pivotal role of the master regulator of *Bordetella* pathogenesis, the sigma factor BtrS, in orchestrating eosinophil-dependent immunomodulation within the context of pulmonary infection. These putative convergent strategies of targeting eosinophils offer promising avenues for the development of novel therapeutics targeting respiratory and other mucosal pathogens.

## INTRODUCTION

Pathogens have co-evolved with their hosts over centuries, specifically selected for their adeptness in dampening host inflammatory responses, thus facilitating infection establishment and persistence. While extensive research has delved into unraveling the intricate mechanisms by which pathogens evade host immune responses, particularly focusing on classical phagocytes (1–8), neutrophils (9–14), epithelial cells (15–21), and adaptive immune cells (12, 22, 23), the exploration of eosinophils (24, 25) remains in its nascent stages. Traditionally associated with parasitic responses or inflammatory pathologies (26–29), eosinophils have been increasingly implicated in bacterial interactions (30–37). Contemporary research now underscores the pivotal role of these cells during mucosal infections (38, 39), offering a fresh perspective on their functional significance.

In our work, we focus on understanding at the molecular and cellular levels what mechanisms *Bordetella* spp. utilize to suppress host immune responses to promote initial colonization, persistence, and transmission (23). In our previous work, utilizing the *Bordetella bronchiseptica* murine model, we discovered a bacterial sigma factor, *btrS* (40, 41) that is critical to suppress host immune responses and delay adaptive immunity (24, 42). Importantly, in the absence of a functional *btrS*, eosinophils became essential to promote adaptive immune response (24, 25), pointing toward a possible mechanism by which *Bordetella* spp. target eosinophils to delay adaptive immune responses.

In this review, we aim to synthesize existing literature linking eosinophils with infections at various mucosal sites, elucidating the diverse functions these cells undertake during different infectious scenarios. Additionally, we will spotlight bacterial effectors known to target eosinophils, either to instigate anti-inflammatory responses or to hyper-activate eosinophils, thereby exacerbating pathology. Finally, leveraging our model system, *Bordetella* spp., we will provide insights into the potential role of eosinophils in exacerbating pathology and fostering the chronicity of infection.

## EOSINOPHIL BIOLOGY AND ROLES IN HUMAN HEALTH

### Eosinophil development: the knowns and unknowns

Before discussing our findings, we have to focus on what is known about eosinophils, their biology, and their functions. Eosinophils were initially described by Paul Ehrlich in 1879 due to their affinity for acidophilic dyes (43). Subsequent research by Cline et al. in 1968 delineated key distinctions between eosinophils and neutrophils, encompassing their distribution, quantity, functions, and specific granules (44, 45). Eosinophils, terminal cells once differentiated (46), migrate to tissue and predominantly inhabit mucosal regions, including Peyer’s patches, gastrointestinal (GI) tract, lungs, mammary glands, and the uterus, where they contribute to tissue, metabolic, and immune homeostasis (46, 47).

While circulating, eosinophils are present in relatively small numbers; however, they exhibit rapid recruitment to tissues in response to specific stimuli (48–50). Tissue-resident eosinophils display remarkable adaptability owing to their plasticity (48–51), which is facilitated by membrane receptors that enable them to respond to complement, chemokines, and cytokines (52). Eosinophil receptors include, but are not limited to, pattern-recognition molecules (53) and pathogen-associated molecular patterns via Toll-like receptors (54), such as several TLRs (53), dectin-1 (55), receptor for advanced glycation end products (56), nucleotide-binding oligomerization domains (57), RIG-I-like receptors (57), or C-type lectin receptors (53). While two populations of eosinophils, circulating and tissue resident, have been established for a long time (58), recent technological developments allowed for a rapid increase in our knowledge and understanding of these complex cell populations. RNA transcriptomic analysis has revealed that there are multiple types of eosinophils. First, they were divided into type 1, a more pro-inflammatory and immunomodulatory phenotype, and type 2, with characteristics similar to the eosinophils isolated during asthmatic processes more associated with Th2 and tissue remodeling phenotypes (59). This classification resembles the type 1 and type 2 macrophages (60). Using single-cell RNA sequencing, five distinct subpopulations of eosinophils have been identified: precursor, immature, circulating, basal, and active eosinophils, with the latter being primarily associated with processes within the GI tract (39). Moreover, active eosinophils play a pivotal role in GI mucosal responses, possessing antibacterial and immunoregulatory functions, including responding to interferon-gamma (36, 59) and interleukin-33 (IL-33) (59). However, the exact mechanisms governing the phenotypes and effector functions of these eosinophil subtypes remain largely unexplored.

Eosinophil granules themselves contain an array of molecules, including cationic proteins, cytokines, chemokines, growth factors, and cytotoxic cationic proteins, which are released via degranulation, also in a stimuli-dependent manner (61, 62). However, the specific molecular patterns of eosinophil responses to different stimuli need to be further investigated. Prominent cationic proteins within eosinophil granules include the major basic protein, eosinophil peroxidase (EPX), and the eosinophil-associated RNases eosinophil cationic protein (ECP) and eosinophil-derived neurotoxin (63). Secretion of these molecules is mostly associated with degranulation. Eosinophil degranulation can occur via piecemeal degranulation (64), exocytosis, or cytolysis (65). The selection processes that dictate eosinophil-secreted components, as well as the mode in which they are released following activation, remain unclear (65), yet these molecules often serve as biomarkers for several eosinophil-related pathologies (66). This suggests that eosinophil activation and the effector functions that follow are spatiotemporally fine tuned, each containing multiple regulatory steps (38). As a result, such intricate multi-step pathways provide many immunomodulatory targets for pathogens to achieve successful infection, which also may be tissue or pathogen dependent.

It is worth noting that mouse and human eosinophils exhibit some differences, with the most apparent one being that human eosinophils contain Charcot-Leyden crystals (CLC) (67, 68), while murine eosinophils do not (69). Nevertheless, fundamental eosinophil functions, cell morphology, effector mechanisms (e.g., degranulation), and basic biology (46) including eosinopoiesis, which is the production or differentiation into eosinophils, remain largely conserved, allowing for the use of animal models that, although not perfect (70), significantly contribute to studying important aspects of eosinophil functionality and development.

### The multifaceted roles of eosinophils

Increased knowledge and understanding of these complex and versatile cells that have been involved in health and disease (26, 43, 71) present an evolutionary enigma (49) about the beneficial or detrimental role eosinophils could possess (43). Eosinophils are notably associated with pathological conditions such as asthma (72), eosinophilic rhinosinusitis (73), eosinophilic esophagitis (74), allergies (28, 71), and fungal infections (75, 76). While the historical focus on eosinophils in infections has predominantly revolved around parasitic (77) and fungal infections (78–82), evidence dating back to the 1970s suggests a direct role of eosinophils in bacterial killing (33, 44, 83, 84). Despite their bactericidal activity appearing seemingly inefficient when compared with neutrophils (33), their ability to successfully phagocytose (45) and kill (44) remains not fully understood. Contrary to the previous perception of eosinophils solely as contributors to disease and helminth infections through proinflammatory responses (85), emerging evidence underscores the complexity of those cells and questions their role during helminth infections as detrimental instead of beneficial, as previously thought (82, 86).

Eosinophils have also been recognized for their roles in lipid metabolism (87–89), vascular homeostasis (90), interactions with the nervous system (91), and cancer immunology (92). Additionally, eosinophils play critical roles in preserving epithelial barrier integrity (93) and releasing cationic proteins that enhance host defense (39, 48). Eosinophils have also been shown to act as regulators of Local Immunity And/or Remodeling/Repair in both health and disease (LIAR hypothesis) (49). Moreover, eosinophils play a critical role in regulatory T cell (Treg) differentiation and tissue homing, mostly via TFG-β (94), altogether highlighting the versatility and multifunctionality of these cells.

To summarize, eosinophil functions could be categorized into four main roles (95). The first includes their terminal effector functions (96), such as phagocytosis (97), eosinophil extracellular trap formation (98, 99), and antibody and complement-mediated cytotoxicity (100). Second, eosinophils participate in tissue repair (28, 63), remodeling (38, 101), and homeostasis by promoting angiogenesis and releasing molecules associated with tissue remodeling (102). The third category is an immune homeostatic role based on their immunomodulatory functions, contributing to immune response polarization (103) of anti-inflammatory or pro-inflammatory responses through early secretion of cytokines and chemokines (104). Finally, the fourth category focuses on the involvement of eosinophils in cell-to-cell communication and signaling, as they share close interactions with epithelial cells via selectins or integrins (43) and they promote immune cell recruitment and activation (105), enhancing antigen presentation (106–110), antigen-specific IgM production (111), and facilitating IgA (112) maintenance through interactions with B cells (48–50).

## *BORDETELLA* SPECIES

The classical *Bordetella* subspecies, encompassing *B. bronchiseptica*, *Bordetella pertussis*, and *Bordetella parapertussis*, are closely related and well-adapted respiratory pathogens capable of infecting humans and/or animals (113, 114), and the genetic mechanisms that regulate host adaptation are very well conserved among classical *Bordetella* spp. (115). *B. bronchiseptica*, infecting a broad host range, is considered the evolutionary predecessor (116–119) to its host-restricted relatives, *B. pertussis* and *B. parapertussis*. As such, *B. bronchiseptica* provides a natural murine infection model that mimics the chronic disease caused by *B. pertussis* infection in humans (120, 121). Notably, wild-type *B. bronchiseptica* can persist in the respiratory tract for extended periods, exceeding at times 56 days post-inoculation (24, 25, 42, 122, 123), facilitating the study of disease pathogenesis in a physiologically relevant setting. The low inoculum needed to cause disease, as low as 5 CFU in mice (124), further underscores the effectiveness of the *B. bronchiseptica* murine model in dissecting the molecular intricacies of the host-pathogen interaction.

Classical Bordetellae exhibit ≥98% nucleotide identity among shared genes (125), as well as nearly identical phosphorylated protein sites throughout their genomes (126) highlighting the level of conservancy of critical functions maintained during the speciation process. The inclusion of several classical *Bordetella* spp. in our work can provide insights into the functional conservation of genes during the speciation process (127).

## ESTABLISHED MECHANISMS OF HOST CELL SIGNALING MANIPULATION DURING *BORDETELLA* COLONIZATION/INFECTION

*Bordetella* spp. can successfully adapt to their environment, owing their success to a variety of evolved mechanisms (113, 128). Numerous virulence genes of classical *Bordetella* are highly conserved at the nucleotide and amino acid levels, regulated by similar two-component systems (TCS) regulatory networks (23). These tightly regulated networks enable the fine-tuned immunosuppression of the host, making *Bordetella* spp. one of the most successful pathogens identified to date. Over the course of infection, classical Bordetellae employ an array of virulence strategies (129), including the utilization of a multitude of bacterial toxins (130, 131), to evade the activation of immune responses initiated by several cell types throughout the host respiratory tract (132).

The infection starts at the epithelium, the first barrier encountered by the bacteria. Initial attachment is mediated by interactions between filamentous hemagglutinin (FHA) (133) and lipid rafts at the host cell membrane (134). Fimbriae (135), pertactin (136), and pertussis toxin (PTX) (137) also contribute to adherence, though to a lesser extent (134, 138). Following attachment to the epithelium, *Bordetella* spp. utilize the adenylate cyclase toxin-hemolysin (ACT) to bind the CD11b/CD18 receptor (139) of target host cells. Both ACT and FHA have been shown to potentiate attachment to the alveolar epithelium (140). During *B. pertussis* internalization by epithelial cells, *Bordetella* spp. impede phagosome acidification to survive intracellularly (19) to escape host immune recognition. To cross the epithelial barrier, ACT causes the disruption of tight junctions, while in parallel causing enhancement of mucus production (141), and elevates IL-6 secretion (142). The T3SS also contributes to colonization of the respiratory tract (127, 143), possibly associated with rapid induction of cytotoxicity to epithelial cells. However, it is important to note that uncontrolled epithelial necrosis *in vivo* has not been observed at similar levels that have been observed *in vitro* (143).

Following translocation across the epithelial barrier, innate immune cells contribute to host defense and become targets of classical Bordetellae to promote the establishment of the infection. *Bordetella* spp. have been shown to induce macrophage cytotoxicity and dampen overall immune signaling cascades (42). PTX binds to the macrophage’s membrane via integrin CR3 (144), and following initial attachment, PTX has similar functions as those performed during epithelial cell infection (145), including inflammasome activation, skewing the phenotype from M1 to M2 (2, 146, 147), or interfering with phagosome maturation (144). While PTX induces macrophage cytotoxicity (7), it has been shown that purified PTX inhibited apoptosis of bone marrow-derived macrophages (145, 148). ACT can induce apoptosis in both macrophages (149) and neutrophils (6, 150), mediated by calcium ion influx (151). But recently, it has been shown that ACT can also inhibit monocyte-to-macrophage differentiation, likely promoting survival of *Bordetella* spp. within the host (152), further highlighting the versatility and complexity of some of these virulence factors.

PTX as well as other virulence factors also promote delayed recruitment of neutrophils (12, 153, 154). Similarly to macrophages, ACT promotes cell death in neutrophils (155), while FHA can promote inhibition of phagocytosis, enhancement of apoptosis, and induction of formation of neutrophil extracellular traps (156). T3SS inhibits neutrophil phagocyte functions *in vitro* (157), recruitment of inflammatory innate cells to the site of infection (158), and induction of innate and adaptive immune responses *in vivo* (159), suggesting a strong association between a functional T3SS and increased persistence of *Bordetella* spp. infection (123, 160).

Overall, *Bordetella* spp. utilize a multitude of context-dependent strategies to manipulate host immune cell signaling pathways, which contribute to initial colonization and establishment of infection. But importantly, how the different functions performed by one toxin are regulated in a cell-specific manner remains unknown.

### The BtrS immunosuppressive pathway: a tool to investigate key host immune players

These toxins aforementioned are not constitutively expressed, and as any other well-adapted organism, *Bordetella* spp. also need to harbor mechanisms to finely tune the expression of genes that are required to adapt to specific host compartments. Since the early investigations that examined *Bordetella* spp. regulatory virulence mechanisms pioneered by established scientists such as Rappuoli et al. (161–163) and Falkow et al. (162, 164–170), many of their successors (171–175) continue to unravel the intricacies behind the great success of this highly transmissible pathogen that continue to rise despite the vaccine being widely distributed for nearly a century. Three regulatory TCS have been extensively investigated (23): BvgAS (170, 172, 176), RisAS (177, 178), and PlrSR (179, 180), yet only some of the genes regulated by these systems have been characterized. In addition to these regulatory TCS, chaperones (181–183), ci-d-GMP (184), sRNAs (172, 185), and sigma factors (23) are only a few additional regulators that finely tune bacterial gene expression to optimize its performance in every environment, including host immune suppression to promote colonization (186), persistence (42), and transmission (187). One gene that is regulated by BvgAS is the extra-cytoplasmic sigma factor *btrS* (40) [also referred to as *brpL* (188)], which was identified (40) and defined (41) as one of the main regulators of the T3SS by Jeffery F. Miller’s group and is the central focus of our laboratory.

**Fig 1:**
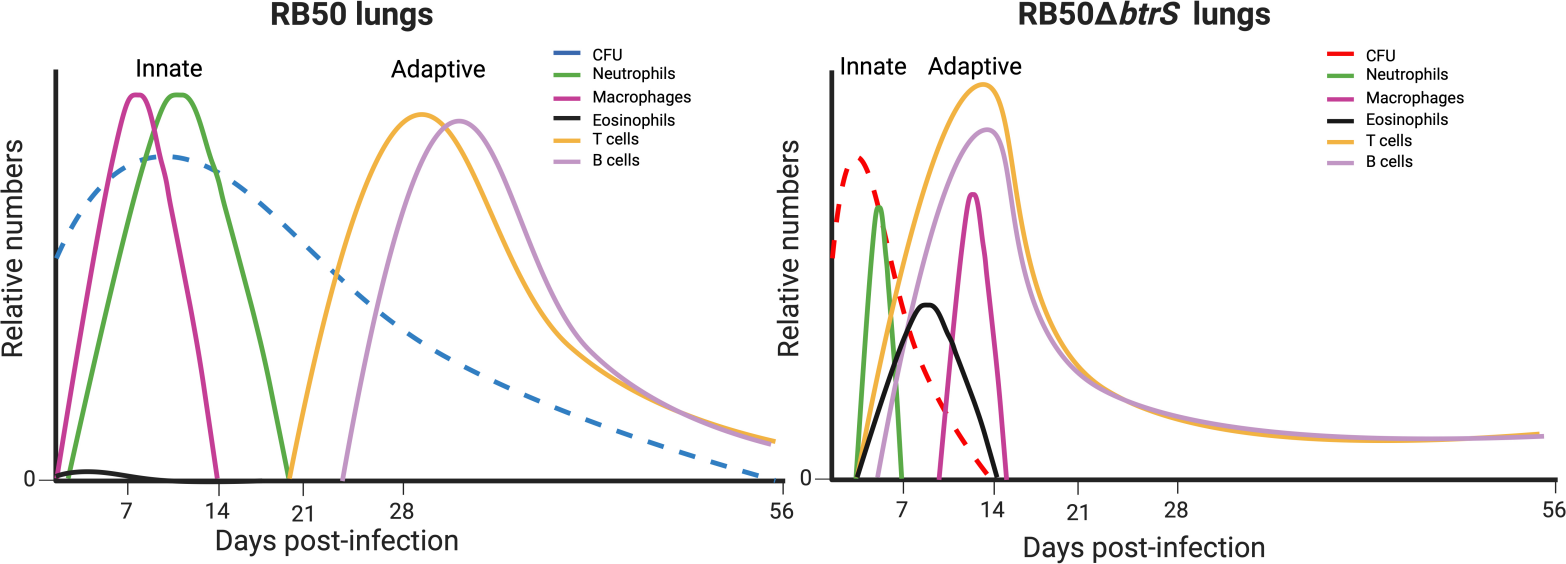
Predicted kinetics of the immune responses to RB50 and RB50Δ*btrS*. The dotted lines indicate lung bacterial burden (blue shows RB50 and red shows RB50Δ*btrS*). Immune cell recruitment to the lungs is shown in green (neutrophils), magenta (macrophages), black (eosinophils), yellow (T cells), and lilac (B cells). Relative numbers are indicated in the Y-axis, while days post-infection are shown in the X-axis.

Our work explored the role of *btrS* using a murine infection model of *B. bronchiseptica*, revealing that *btrS* regulates many known and unknown virulence factors beyond the T3SS (42). In the absence of *btrS*, murine *B. bronchiseptica* infection clears more rapidly from the respiratory tract due to a stronger and more effective immune response (42) (Fig. 1). The immunity generated by the *btrS*-null mutant results in long-lasting sterilizing immunity in the murine model that lasts up to 15 months in the lungs and 7 months in the nasal cavity (189). Importantly, our results indicate that early adaptive immune responses generated by infection with the *btrS*-null strain required eosinophils (25). Combining RB50, a wild-type bacterium that is highly successful at suppressing host immune responses, and RB50Δ*btrS,* which is rapidly clear and generates a profound immune response overpassing convalesce immunity (189), provides a unique opportunity to better understand the molecular mechanisms that underlie pathogenic success, which we interpret as increasing persistence and enhancing transmission. Our data point toward eosinophils as drivers of efficient adaptive protective immune responses after *btrS*-null infection (24, 25); however, this role for eosinophils is not noted during infection with the RB50 strain or any other *Bordetella* spp. or many respiratory bacteria, suggesting that *Bordetella* spp., and possibly other respiratory pathogens, are so well adapted to suppress eosinophil effector function that the role of these cells is masked.

## EOSINOPHILS AS BACTERIAL TARGETS TO FACILITATE *BORDETELLA* PERSISTENCE

Utilizing the *btrS*-null mutant revealed a role for eosinophils previously unnoted. Although some *Bordetella* spp. have been indirectly associated with eosinophils in many studies over the years, this is a logical observation, as epithelial cells and eosinophils exhibit intimate crosstalk and elaborate signaling networks (102). As early as 1969, the utilization of a *B. pertussis*-treated murine model was reported for assessing eosinophil cell responses to antigenic challenges (190), although no effects on eosinophils was observed, this primed the field and other studies of hypersensitivity in *Bordetella* followed (190–192). *Bordetella* spp. infections have also been recognized as stimuli for triggering asthmatic and allergic events (192). To further intertwine *Bordetella* and eosinophils, both acellular and live-attenuated pertussis vaccines have been associated with preventing asthma exacerbations (193–198).

One of the drivers of asthmatic airway remodeling is sphingosine-1 phosphate (S1P) (199). During *B. pertussis* infections, *in vivo* inhibition of sphingosine-1 phosphate (S1P) signaling in murine models reduces *Bordetella* pathology without affecting bacterial burden (200). S1P activation promotes airway remodeling, recruitment of eosinophils expressing S1P receptors, and induction of other Th2-related cells (61) and increases smooth muscle mass (201) during asthmatic processes. This is coupled with epithelial-mesenchymal transition, which contributes to elevated lung resistance and fibroblast activation and results in hyperresponsiveness (202), potentially establishing a mechanistic link between *Bordetella* infections and the asthma-like pathologies, symptoms, and long-lasting consequences that are present during and after pathogen clearance.

Similarly, low concentrations of vasoactive intestinal peptide or VIP mediate asthma pathology (203) following interaction with one of its receptors, VPAC2. Investigating if the VIP/VPAC2 axis can interfere with *Bordetella* spp. infectious process, our result revealed that the three classical *Bordetella* spp. utilize the T3SS to stimulate VPAC2 signaling and promote persistence in the lower respiratory tract (123). In the absence of functional VPAC2 signaling, *Bordetella* spp. are cleared more rapidly. Furthermore, our results indicate that *Bordetella* spp. utilize the T3SS to activate VPAC2 signaling, leading to increased persistence, especially in the lower respiratory tract.

Indirect evidence for a potential role of Th2 inflammation in *Bordetella* spp. infections has been shown using a rabbit model of *Bordetella bronchiseptica* co-infection with helminths. Helminths are known to activate eosinophils and induce Th2 responses. During rabbit co-infection of *B. bronchiseptica* and helminths, the results showed enhanced shedding and transmission of *Bordetella* (204). Although this evidence is correlative, it points to a relationship between *Bordetella* spp. and eosinophils where we envision *Bordetella* spp. suppress eosinophil pro-inflammatory functions and stimulate tissue remodeling associated with the classical bronchoconstriction observed in pertussis patients (205).

**Fig 2:**
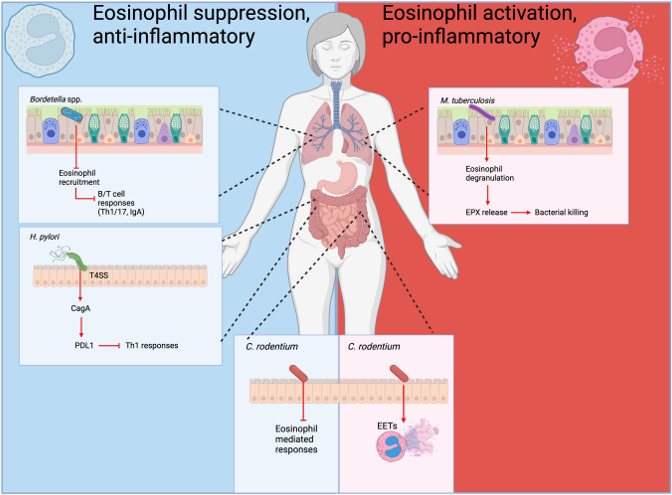
Multifaced roles of eosinophils. Eosinophils can be considered into two categories, more anti-inflammatory phenotype (blue) associated with bacterial persistence or more pro-inflammatory (red) where eosinophils actively contribute to bacterial killing during infection.

Taking advantage of our *in vivo* murine infection model encompassing wild-type *B. bronchiseptica* (RB50) and a mutant strain lacking *btrS* (RB50∆*btrS*) (25), we investigated the contribution of eosinophils to the generation of adaptive immune responses during infections with the *btrS*-null strain. Our results revealed that in the absence of *btrS*, lung eosinophils promote recruitment of B/T cells to the site of infection, with T helper (Th) lymphocytes skewed toward Th1/17 phenotypes and increased production of IgA from B cells. Furthermore, this immune response was mediated by eosinophil-derived chemokine (C-motif) ligand 1 (XCL1) (24). Together, these recruited cells formed an inducible bronchus-associated lymphoid tissue (iBALT) with eosinophils within or proximal to these aggregates. In the absence of eosinophils, B/T cell recruitment, IgA production, and iBALT formation were suppressed, suggesting a role for eosinophils as mediators of protective adaptive immune responses (24) (Fig. 2).

This discovery may be the first step in elucidating the hitherto unrecognized effector functions of eosinophils in response to other respiratory pathogens. However, this discovery was made using our *btrS*-deficient *B. bronchiseptica* strain; the role of eosinophils during wild-type infection is drastically different, with no effect for eosinophils noted at all, suggesting that *B. bronchiseptica* is so well adapted to suppress eosinophil activity and that the presence or absence of eosinophils has no apparent effect on bacterial persistence.

Overall, the evidence provided in this section underscores a sound connection between the immunomodulatory roles of *Bordetella* spp. and eosinophils, posing the bigger question of whether these immune cells are direct bacterial targets or somehow instigators of bacterial infections. These observations collectively highlight the intricate interplay between *Bordetella* spp. and eosinophils, shedding light on potential therapeutic approaches. Our proposed model postulates that *Bordetella* spp. infections initiate asthmatic-like processes, and with a higher frequency of infections, the inflammation becomes chronic (206, 207), exacerbating associated symptoms and promoting anti-inflammatory microenvironments that allow for bacterial persistence. These strategies might also be conserved among other respiratory pathogens, as similar connections have been identified, revealing the hidden role of these cells as mediators of immune responses.

## BACTERIAL EXPLOITATION OF EOSINOPHILS BEYOND *BORDETELLA*

### Eosinophils in GI: *Helicobacter pylori* infection

Many of the functions and roles eosinophils play during mucosal infections have long been intriguing. In the GI tract, research suggests a critical role for eosinophils during *Helicobacter pylori* infections (208) and the involvement of these cells to facilitate persistence (209). *H. pylori* outer membrane vesicles promote eosinophil degranulation, and the vacuolating cytotoxin VacA enhances the secretion of EPC. Moreover, this effect is also mediated by epithelial cells, highlighting the crosstalk between epithelial cells and eosinophils during GI infections (210). It has been shown that the *H. pylori*-derived peptide, HP(2-20), induces eosinophil recruitment to the superficial layer of the mucosa, leading to eosinophil activation. This facilitates wound healing via eosinophil-derived vascular endothelial growth factor A and tumor growth factor beta, which promote tissue remodeling and integrity (32, 211, 212). Eosinophils also modulate T cell responses during *H. pylori* infection utilizing the type 4 secretion system to promote the secretion of the anti-inflammatory molecule, program death ligand 1, which dampens immune responses (37). The fine-tuned relationship between *H. pylori* and eosinophils is becoming more complex as we better understand the cross-signaling between *H. pylori* and eosinophils. *H. pylori* harbor chemoreceptors, one being encoded by the *tlpA* gene. *tlpA* senses beneficial molecules such as arginine, fumarate, and cysteine, which lead to increased growth showed through an *in vivo* infection model. Johnson et al. recently showed that *tlpA* suppresses eosinophil recruitment at later times post-infection which might correlate with the increased persistence observed during infection with the wild-type *H. pylori* (213), as previous evidence has shown that eosinophil depletion enhances the clearance of *H. pylori* infection, amplifying Th1 responses (37–39). Moreover, during *H. pylori* infection, deregulation of a proliferation-inducing ligand (APRIL) has been associated with tumorigenesis, and the population of APRIL-producing eosinophils infiltrated tumors in higher numbers in patients with *H. pylori* infection, suggesting a pro-tumorigenesis role for eosinophils in *H. pylori*-infected patients (214).

Tissue-resident eosinophils also play a critical role in maintaining tissue homeostasis (47), communicating closely with the gut microbiota (215), potentially impacting the number of neonatal infection cases of *H. pylori* (38). Furthermore, correlative evidence has suggested that *H. pylori* infection protects against eosinophilic esophagitis (216, 217) and reduced responses to allergic asthma (218, 219), contributing to a protective role of neonatal *H. pylori* infection in mitigating the severity of asthma and allergic airway inflammation, possibly via Treg-mediated mechanisms (218). However, it is crucial to note that *H. pylori* infections can also trigger autoimmunity, a phenomenon requiring further investigation (Fig. 2).

### Eosinophils in GI: *Citrobacter rodentium* infection

There are other bacterial GI pathogens that also show close interplay with eosinophils. Recent findings indicate that eosinophil-deficient mice show increased susceptibility to *Citrobacter rodentium* infection, accompanied by heightened Th17 responses and exacerbated disease pathology (39). Similarly, selective deletion of ATG5 in the eosinophil lineage resulted in impaired eosinophil effector function, resulting in faster clearance of *C. rodentium* infection (220). Eosinophils effectively kill *C. rodentium* both *in vitro* and *in vivo* through eosinophilic cationic proteins associated with extracellular traps (37) and can present antigens via MHC-I^39^, underscoring their critical roles during bacterial infections in the GI tract.

### Eosinophils in the skin: *Staphylococcus aureus*

Atopic dermatitis is one of the most common eosinophilic disorders generally associated with recurrent *Staphylococcus aureus* skin infections (221); eosinophils have also been shown to be involved in immune responses and allergic reactions to *S. aureus* (96). When exposed to *S. aureus*, eosinophils produce superoxides, release granule-derived molecules (e.g., ECP), and exhibit bactericidal activity for infection clearance (222). The *S. aureus* peptidoglycan has been identified to activate eosinophil recruitment (223). There is additional evidence of eosinophils being a direct target for promoting an anti-inflammatory environment during *S. aureus* infection *in vivo* (224) (Fig. 2).

### Eosinophils in the lung

We have discussed the role of eosinophils during *Bordetella* spp. infections (24), but this is not the only lung pathogen that has been associated with eosinophils. *Mycobacterium tuberculosis* (Mtb) has also been closely associated with eosinophils (225), and persistent eosinophilia in patients with tuberculosis has been commonly reported (225, 226). Eosinophils are a critical component of the immune response to generate protection against Mtb infection (227). It has been shown that decreased numbers of peripheral eosinophils correlate with disease severity caused by Mtb infection. Moreover, eosinophils are present on the rim area of granulomas which actively secrete EPX and diffuse to the central necrotic core area found in pulmonary lesions. Eosinophils do not appear to internalize Mtb, but rather, these cells degranulate and secrete EPX, which serves to aid in controlling bacterial growth. Importantly, these findings were obtained in separate animal and *Mycobacterium* spp. models, indicating a high level of conservation in this type of immune response (228). Further highlighting the important role eosinophils play during Mtb infection, they are one of the first cells to be recruited to the infection site and rapidly interact with tissue-resident macrophages. Tissue-resident macrophages can contain Mtb, and Mtb-infected cells upregulate the oxysterol-producing enzyme Ch25h, facilitating binding to the oxysterol receptor GPR183 expressed on circulating eosinophils and triggering migration to the lungs during early stages of pulmonary Mtb infection (229). In conclusion, eosinophils play a crucial role in the immune response to *M. tuberculosis* infections, with evidence suggesting their involvement in controlling bacterial growth within pulmonary lesions. Their early recruitment to the site of infection and interaction with infected cells highlight their importance in orchestrating protective mechanisms against Mtb, underscoring the significance of understanding their function in tuberculosis pathogenesis and potential therapeutic interventions (Fig. 2).

## PERSPECTIVES

For long, eosinophils have been considered the primary responders to parasitic infections as well as the main responsible cells for asthmatic and allergic reactions. However, cumulative evidence has been pointing toward a more elegant and subtle role for these cells in the coordination of immune responses to other stimuli and pathogens. Cumulative evidence indicates eosinophils contribute to viral (109, 230–232) and fungal (233–235) infections, although we have not focused on these aspects during this review. In the context of bacterial infection, it has been shown that bacteria utilize specific toxins or secreted effectors to establish infection in the epithelia, cross the epithelial barrier, and manipulate phagocytes and neutrophils to redirect the fate of host immune responses toward mechanisms that will facilitate establishment of infection and persistence. However, despite our deep knowledge of some bacterial mechanisms, the discovery of the *btrS*-immunosuppressive pathway has revealed that our knowledge represents only the tip of the iceberg when discussing bacterial immunomodulation of hosts.

One of the main dogmas challenged with the *btrS*-null mutant requires the revision of the convalesce immunity as a gold standard. Our previous work revealed that in the absence of *btrS*, not only recruitment of adaptive immune cells is accelerated leading to more rapid clearance of infection (42). Furthermore, we have observed that the immunity provided by prior challenge with *btrS*-null is more robust and long lasting than that provided by prior infection (189). But why to be surprised if it is well accepted that bacteria suppress host immune response? Then, it would make sense that infection with a mutant that cannot suppress, manipulate, or evade immune responses will result in better immunity.

Another innovative result focuses on the evidence that eosinophils are required for adaptive immune responses to infection with the *btrS*-null mutant. These results encompass multiple concepts; on one side, this suggests that eosinophils might be required for protective immune responses and that in fact, these cells contribute to the robust B and T cell responses observed after *btrS-*null infection. Secondly, the fact that a function for eosinophils has not been previously noted might suggest that well-adapted pathogens have been selected for their ability to suppress eosinophil effector functions to promote their survival and persistence within the host. This will be supported by our findings with the *btrS*-null mutant (24) as well as the previously published evidence on other models such as the *H. pylori* infection model (38). Could it be that pathogens that cause chronic pathologies share a convergent core strategy of targeting eosinophils to promote persistence? What role could eosinophils serve for obligate intracellular bacterial pathogens? Could it be possible that allergies and asthma are the result of pathogens manipulating eosinophils during infections leading to hyper-reactivity of these cells? In fact, the evidence herein presented shows how at different mucosal sites a myriad of pathogens use convergent strategies to target these cells and promote degranulation to initiate inflammation leading to allergic states of disease or inducing secretion of anti-inflammatory signaling cascades that enhance pathogen persistence leading to chronic infections.

**Fig 3:**
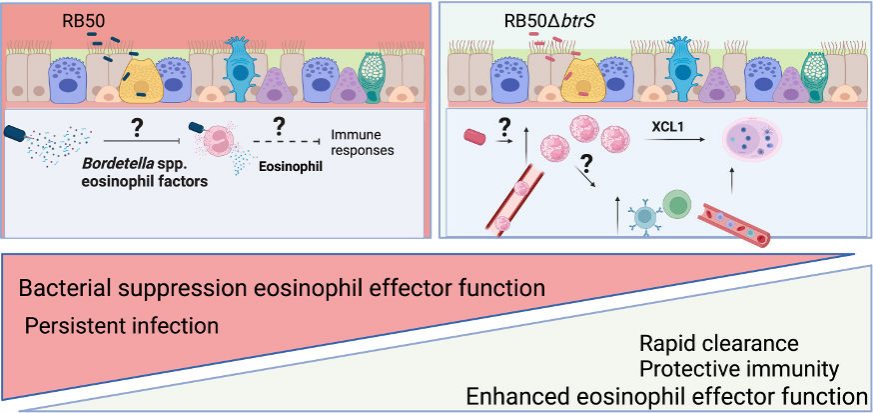
Predicted role of eosinophils during *Bordetella* spp. infections. *Bordetella* spp. secrete “*Bordetella* eosinophil factors” that will suppress eosinophil effector function leading to persistence. Deletion of the bacterial sigma factor *btrS* results in no suppression of eosinophils. In this context, eosinophils promote adaptive immune responses, formation of germinal centers in the lungs, and rapid clearance of infection.

How eosinophils interact with mucosal pathogens is an open frontier of discovery that has decades of work to be done by many fields of researchers. Using current knowledge about how *Bordetella* spp. modulate host immune cells and barriers spanning over the whole infection life cycle, as well as illuminating current understandings of the role of eosinophils during infection, our future work aims to identify a central role for eosinophils during this process. We envision that *Bordetella* spp. harbor specific virulence mechanisms or factors that target eosinophils to promote persistence as well as pathology during the infectious process. Furthermore, we expect these strategies will be conserved among different *Bordetella* spp. Here, we propose that these once underappreciated eosinophils are not only an integral but also common target for establishing persistence and they may serve as the missing link to other enigmatic pathogens that have evolved similar modes of action to those of classical Bordetellae (Fig. 3).

By unraveling the complexities of eosinophil-pathogen interactions, we can pave the way for innovative therapeutic interventions targeting these immune cells and enhance our ability to combat bacterial infections effectively. Altogether, appreciation of these findings only highlights the need for a better understanding of how eosinophils function during bacterial infections and how these mechanisms can be targeted for therapeutic and preventative measures against infection, transmission, and also disease severity induced by these pathogens. Using our *Bordetella bronchiseptica* and *Bordetella pertussis* murine models, our work will focus on unravelling the cellular and molecular mechanisms by which *Bordetella* target eosinophils during infection as well as the role of eosinophils during *Bordetella* infections. This twofold approach will provide a novel view for eosinophils during the infectious process, and we expect that our results would provide a better understanding on the inter-signaling between *Bordetella* and eosinophils during infection.
